# Economic losses associated with respiratory and helminth infections in domestic pigs in Lira district, Northern Uganda

**DOI:** 10.3389/fvets.2023.1198461

**Published:** 2023-06-16

**Authors:** Peter Oba, Michel Dione, Frank N. Mwiine, Barbara Wieland, Joseph Erume, Emily Ouma

**Affiliations:** ^1^International Livestock Research Institute (ILRI), Kampala, Uganda; ^2^National Agricultural Research Organization (NARO), Abi Zonal Agricultural Research and Development Institute (Abi ZARDI), Arua, Uganda; ^3^International Livestock Research Institute (ILRI), Dakar, Senegal; ^4^College of Veterinary Medicine, Animal Resources and Biosecurity (COVAB), Makerere University, Kampala, Uganda; ^5^Institute of Virology and Immunology, Mittelhäusern, Switzerland; ^6^Department of Infectious Diseases and Pathobiology (DIP), Vetsuisse Faculty, University of Bern, Bern, Switzerland

**Keywords:** average daily weight gains (ADGs), economic losses, respiratory pathogens, pigs, *Ascaris* spp., Uganda

## Abstract

This study sought to quantify direct economic losses due to respiratory and gastrointestinal (GI) helminth infections in domestic pigs in Uganda. In a longitudinal study design with repeated measures, farm visits were made at 2 month intervals from October 2018 to September 2019. Weaner and grower pigs (*n* = 288) aged 2–6  months from 94 farms were sampled. The pigs were monitored for growth and screened for exposure to four important respiratory pathogens: porcine circovirus type 2 (PCV2), porcine reproductive and respiratory syndrome virus (PRRSv), *Mycoplasma hyopneumoniae* (*M. hyo*), *Actinobacillus pleuropneumoniae* (*App*) using ELISA tests. Farm management practices were recorded and used to generate management level scores. Treatment expenses incurred were recorded throughout the study. A mixed effects model was fitted to quantify effects of respiratory and helminth infections on average daily weight gains (ADGs), with farm and pig as random effects. Analysis of variance (ANOVA) was used to determine differences in mean treatment costs by farm management standard. Financial losses were estimated from average carcass dressing percentage, ADG reductions during fattening (200  days). Results showed a grower pig in a given farm exposed to PRRSv and *Ascaris* spp. had significantly lower ADG by 17.10 gr/day and 16.80 grams/day respectively, compared to a similar unexposed pig (*p* < 0.05). Mean treatment costs per pig declined significantly with increase in management standard scores (MSS), from USD 1.13 per pig in MSS 1 (poor management) farms to USD 0.95 for MSS 3 (better management) farms (*p* < 0.05). We show that monetary losses due to PRRSv and *Ascaris* spp. infection amounted to USD 6.6 ± 2.7 and 6.50 ± 3.2 (Mean ± SEM) per pig, respectively during 200 days of fattening. This study strengthens evidence that improving management practices to reduce infections mitigates economic losses. To guide interventions, further studies are required to unravel the full extent of indirect economic losses.

## 1. Introduction

Respiratory diseases cause significant economic losses to swine producers globally due to reduced productivity, increased production costs and reduced market opportunities (1–3). Economic losses vary considerably between countries due to differences in production systems, study methodologies applied and types of pathogens involved, as the interactions produce varying levels of disease severity.

Economic losses due to swine diseases result from mortalities, reduced weight gains (4), poor reproductive performance, negative effects on feed conversion and increased costs of treatment (5–7). Reduced market value of sick animals represents indirect disease effects, which producers often encounter. Other losses result from carcass condemnations due to lung lesions or reduced carcass quality (8, 9). Previous studies have shown that multiple infections increase the severity and duration of clinical disease (10, 11). To provide a framework for design of interventions at the pig production node of the value chain, it is necessary to quantify possible production losses. This information is useful for producers and extension services to support investment decisions to improve herd profitability.

The disease burden is higher in developing countries due to poor biosecurity practices and poor nutrition, suggesting the extent of possible economic losses may be considerable (12–14). In Uganda, despite existence of opportunities for improving livelihoods (15, 16), the pig sector faces many constraints among which are endemic diseases. Much research focus has been on African swine fever (ASF), a major disease of devastating economic consequences (17, 18). However, other respiratory pathogens of significant economic losses in pig production (PCV2, PRRSv, *M. hyo*, *App* and *Streptococcus suis*) have been reported (19–21).

In Uganda, economic losses associated with pig respiratory diseases are largely unknown, as no such studies exist. This study aimed to quantify economic losses (average daily weight gains, ADGs) due to exposure to selected respiratory pathogens and GI parasites. Furthermore, it sought to determine effect of management level on treatment costs.

## 2. Materials and methods

### 2.1. Study area

**Figure 1:**
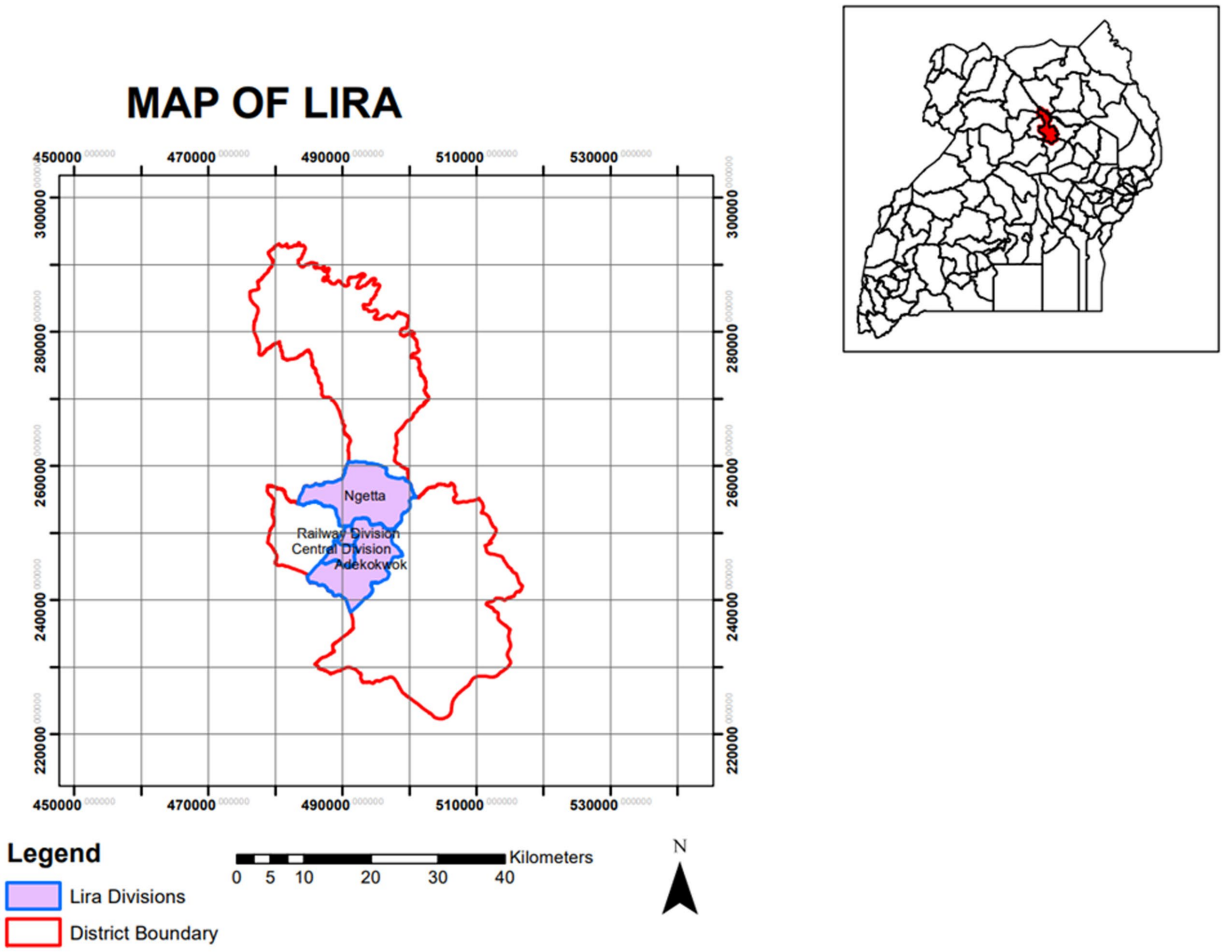
Map of Lira district in Uganda showing study areas.

This study was conducted in four selected subcounties of Lira district, mid-northern Uganda, characterized by predominantly smallholder production systems. The selection of subcounties was based on the dominant value chain domain, as described in a previous study (22). In this area, pigs are raised by mainly tethering in rural areas or housed as in urban or Peri-urban settings (23, 24). In these systems, farmers keep few pigs, usually 1–3 sows and 3–5 weaners for farrow to finish systems. Further description of the study area and selection of subcounties and villages for this study is detailed in (22). Anthelmintics and/or antibiotic treatments were given to pigs as preventive or curative measures in some farms. Figure 1 below shows a map of Lira district Uganda where the study was conducted.

### 2.2. Study design

The study was done as a prospective cohort study from October 2018 to September 2019. Live weight measurements and blood sample data was collected at 2 month intervals. We targeted all farms with ≥2 weaner or grower pigs during the study, to reduce losses to follow up. A rolling recruitment procedure was used to enroll pigs for the study, because not all 2 pigs in each farm were available initially. For pigs which died or were sold, new pigs of approximately same weight and age from the same herd were enrolled.

### 2.3. Sampling of farms and pigs

We monitored farms in the urban and Peri-urban settings only because this was where we could find enough accessible confined pigs. We sampled only confined pigs (housed or tethered) because roaming pigs were difficult to capture and monitor. In each farm, we targeted to sample at least 2 weaner (2–3 months) and/or grower pigs (4–6 months). From a sampling frame of a list of pig keeping households obtained from the district veterinary office, random sampling was done. Informed consent was obtained from pig owners to participate in the study and find out their willingness to keep pigs for ≥6 months. In each farm, weaner and grower pigs were randomly sampled. Enrolled pigs were identified by ear tagging and data recorded at the onset and on subsequent visits.

### 2.4. Sample size determination

The following assumptions were made based on a study by Carter et al. (25): a normal uninfected weaner pig (from 2 months) grows from 8 to 30 kg (5 months). So, average daily gain (ADG) is 147 gr/day. An infected pig grows from 8 kg to 20 kg in 5 months; so ADG is 80 gr/day (Dohoo, 2018, personal communication). The difference in ADGs is 147–80 = 67 gr/day. Assume a standard deviation of 70 gr/day (*s* = 0.06), estimated common variance for the two groups, *σ*^2^ = 0.49. To quantify effects of pathogen exposure on ADGs, a sample size for repeated measures design was calculated from Eq. (1) (26):


(1) $$n=2{\left(Z_{\alpha /2}+Z_{\beta }\right)}^{2}\left(1+\left(n-1\right)\rho \right)/n{\left[\left(\mu_{1}-\mu_{2}\right)\sigma \right]}^{2}$$

where *Z*_*α*/2_ is the standard normal deviate for *α* = 1.96, *Z_β_* = −0.84, and *μ*_1_ − *μ*_2,_ the detectable difference in mean ADGs between unexposed and exposed pigs, taken to be 67 gr/day (*d* = 0.067) and the number of time points, *n* = 3. The assumed correlations of repeated measures, *ρ* = 0.6. Using this equation, the total required sample size (*n*) for the study, *n* = 197 pigs.

### 2.5. Data collection

#### 2.5.1. Measurement of live weights, body condition scores, and clinical disease scores

Excel sheets were designed and used to record data at farm and pig level. Data was collected at 2 month intervals in a repeated measure longitudinal study design, with replacements of pigs if they were sold or died. Data on farm management variables likely to be associated with infection and growth in pigs were captured. Data collected included rearing method (housed or tethered), pig age, sex, breed (local vs. improved), live weight (kg) measured using a *HiWeigh^®^* BSR5300 weighing scale (*Shanghai, China*), feed quality grade used based on crude protein (CP) content: grade 1 = sole grazing only (2–7%), grade 2 = sole grazing and maize bran (7–10%), grade 3 = maize bran and swill (11–14%), and grade 4 = compounded (15–18%) feed (27, 28). Body condition scores (BCS) were scored as follows: 1 = very emaciated, 2 = thin, 3 = moderate, 4 = fat, and 5 = very fat.

To estimate direct production costs, treatment expenses throughout the study were recorded. Drug treatments were recorded per herd and divided by herd size to estimate average cost per pig during the 60 day sampling interval. These included types and cost of drugs bought (dewormers, antibiotics) and veterinary fees. Herd size and perceived herd value (based on farm-gate prices) were recorded at the start and at each visit. Expenses and value of pigs were recorded in Uganda shillings. Fixed and other variable costs (e.g., feeds) were omitted because of lack of reliable records.

#### 2.5.2. Blood sample collection and analysis

Pigs were monitored for exposure to 4 respiratory pathogens by blood sampling at each visit. Larger pigs were restrained with a metallic pig catcher (*Model BZ002, MG^®^ Livestock, Shandong, China*), while smaller pigs were restrained by hand. With a pig properly restrained, blood was collected from the cranial vena cava or jugular vein, using a 21G, 1.5″ needle into 5 mL plain BD^®^ vacutainer tubes. The tubes were labeled with pig details and placed in icebox at 4–6°C. Shortly after collection, samples were delivered to Lira district veterinary laboratory for temporary storage. Blood samples were left to stand at room temperature (20°C) overnight and serum harvested the next day into 2 mL cryotubes (*Sarstedt^®^, Germany*), labelled and stored in a fridge at −20°C until testing.

#### 2.5.3. Serological analysis

At the time this study was conducted, no pig vaccines against any respiratory disease were available on the market, implying no vaccination was done. Thus, a serological test that turns out positive is interpreted as a likely result of natural infection. This study monitored pigs prospectively for growth and exposure to four key respiratory pathogens: PCV2, PRRSv, *M. hyo* & *App*. While both PRRSv genotypes were detected in Uganda, type 1 was more prevalent (29). The ELISA test kit was designed to detect both PRRSv genotypes. Sera were screened using specific ELISA assays according to manufacturer’s instructions for each pathogen. Suspect samples were re-tested. Table 1 shows a summary of ELISA test characteristics.

**Table 1:** Summary of ELISA test characteristics.

Pathogen	ELISA test kit manufacturer	Test sensitivity (*Se*) and specificity (*Sp*)	Cut-off sample to positive ratios (S/P%)
PCV2	Krishgen Biosystems, India	*Se* 92.0%, *Sp* 94.0%	positive ≥ 0.2; negative < 0.2
PRRSv	Krishgen Biosystems, India	*Se* 94.0%, *Sp* 94.0%	positive ≥ 0.2; negative < 0.2
*M. hyo*	IDDEXX, Westbrook, Maine, United States	*Se* 85.6%, *Sp* 99.6%	positive > 0.4; negative < 0.3
*App*	IDDEXX, Westbrook, Maine, United States	*Se* 97.8%, *Sp* 100%	positive ≥ 0.5; negative < 0.4

#### 2.5.4. Faecal sample collection and analysis

At each farm visit, faecal samples (~5 gr) were collected from the rectum of each pig using gloved hands into 5 mL plastic containers, labelled and placed in ice box at 4°C. Samples were screened for presence or absence of *Strongyles* spp. and *Ascaris spp.* helminths at the College of Veterinary Medicine, Animal Resources and Biosecurity (CoVAB), Makerere University. Eggs were hatched (Baermann’s method) and larvae used to identify helminth species (30).

#### 2.5.5. Data analysis

Data was cleaned, coded, and analyzed using R Statistical software (31). To quantify effects of respiratory infection(s) on ADGs, a mixed effects (MEM) model was fitted (with farm and pig as random effects) which considers the multilevel structure of the data (pigs nested within farms & observations within pig). An individual pig was the unit of analysis, in which infection was defined as a positive ELISA test to any one respiratory pathogen. The average weight gain (ADG gr/pig/day) was computed using Eq. (2) below (32).


(2) $$\begin{array}{l} ADG\;\left(gr/day\right)=\;\;\left(Liveweight\;\left(kg\right)\;presentvisit\right)\\ \;\;\;\;\;\;\;\;\;\;\;\;\;\;\;\;\;\;\;\;\;\;\;\;\;-\left(Liveweight\;\left(kg\right)\;at\;previousvisit\right)\\ \;\;\;\;\;\;\;\;\;\;\;\;\;\;\;\;\;\;\;\;\;\;\;\;\;/Timeinterval\;\left(days\right) \end{array}$$

The following variables were used to fit a mixed effects model: pig age, pig sex, pathogen serostatus, parasite infection and body condition score (BCS). The R packages “*lme4,” “lmer,” “lmerTest,” “reghelper”*, and *“jtools”* (33) were used to fit the model. The mixed effects model for mean ADG $Y_{ij}$ for an individual pig *j*, in a given farm *i,* was estimated from Eq. (3):


(3) $$ADG_{ij}=\beta_{0}+\beta_{1}X_{1}+\ldots \ldots \beta_{k}X_{ki}+\mu_{farm\left(i\right)}+\varepsilon_{ij}$$

where *β*_0_, *β*_1_ are the fixed effect coefficients for the intercept and slope respectively; *X*_*i*,_ *X_ki_* are the fixed effects regressors (*i* = 1, 2, …), *μ*_farm(j)_ is a random effect of farm *i*, and the residual error term, $\;\varepsilon_{ij}$ ~ *N* (0, τ ) assumed normally distributed. Random effect terms were included for nested design of pigs within farms, which allows for varying intercepts and slopes.

As farm management practices directly or indirectly affect pathogen exposure, variables known or suspected to influence exposure rates and weight gains were captured. A pig *housing index* (hi) was derived from the method of (34) used as a proxy for poverty, but with modification. The index represented aggregated individual scores of pig house components based on materials used on the floor, wall, and roof. The higher the housing index, the better the quality of housing (range 1–24). Farm management practices: routine drug use, floor hygiene level, access to extension services and whether farmers isolated sick pigs were used to generate a management standard score (MSS). Analysis of variance (ANOVA) was used to establish differences in treatment costs between 3 farm management levels at *p* < 0.05. Financial losses were estimated from average carcass dressing percentage, ADG reductions during fattening, which in Uganda’s smallholder settings is taken to be 200 days (from data). The model was fitted using Residual Maximum Likelihood (REML) estimation procedure and selection was done using *Akaike Information Criterion* (AIC). Model diagnostics were evaluated by plotting a graph of fitted values versus residuals.

## 3. Results

Table 2 shows 288 pigs from ninety-four (94) farms that were sampled and monitored. Male household heads constituted 53.4% (*n* = 53), while females accounted for 43.6% (*n* = 41).

**Table 2:** Descriptive summary statistics by farm location, education level, and house type.

Characteristics	Category	No. of farms sampled (*n* = 94)		Totals (%)
		Males (%)	Females (%)	
Location (Subcounty)	Adekokwok	28 (30.10)	25 (26.80)	53 (56.38)
	Central division	6 (6.40)	2 (2.10)	8 (8.50)
	Ngetta	7 (7.40)	10 (10.70)	17 (18.10)
	Railways	12 (12.9)	4 (4.30)	16 (17.00)
Farmer education level	Never attended	1 (1.06)	3 (3.20)	4 (4.25)
	Primary	19 (20.21)	23 (24.46)	42 (44.68)
	Secondary	14 (14.90)	8 (8.51)	22 (23.40)
	Graduate	15 (15.95)	7 (8.51)	22 (23.40)
	Post-graduate	4 (4.25)	0 (0.00)	4 (4.50)
Pig house type	Housed	27 (28.72)	24 (25.53)	51 (54.25)
	Tethered	26 (27.90)	17 (18.08)	43 (45.75)
	Totals	53 (56.38)	41 (43.62)	94 (100.0)

Results showed significant correlations between live weights and ADGs (*r* = 0.37, *t* = 11.8, *p* < 0.000), live weights and visits (*r* = 0.50, *t* = 16.9, *p* < 0.000), and between live weights and pig age (*r* = 0.68, *t* = 27.7, *p* < 0.000). The mean live weight (Mean ± SD) of pigs in this study was found to be 18 ± 6.9 kg for pigs of 2–4 months, 29.7 ± 11.8 kg for pigs of 5–8 months and 47.4 ± 15.5 kg for pigs of 9–15 months. This suggests pigs were generally underweight for their age, as reflected by poor body condition scores (from data). The value of pigs sold, and mortality observed varied between subcounties (Table 3).

**Table 3:** Summary of farm characteristics: herd size, pigs sold, number, and value of dead pigs.

Variable	Subcounty				Totals
	Adekokwok	Central div.	Ngetta	Railways	
No. of farms sampled (%)	53 (56.4)	8 (8.5)	17 (18.1)	16 (17.0)	94 (100)
No. of pigs sampled (%)	160 (55.6)	26 (9.0)	66 (22.9)	36 (12.5)	288 (100)
Herd size per farm (Mean ± SEM)^*^	5.2 ± 0.36^a^	11.8 ± 1.09^b^	15.9 ± 3.47^c^	14.8 ± 2.15^c^	9.3 ± 1.77
Value of sold pigs (median)^¶^	69.10	256.90	555.25	421.27	410.00
No. of pigs sold/farm (median)	2	5	13	8	7
Price/pig sold (mean)^¶^	34.55	51.38	42.71	52.66	45.32
Percent of pig deaths/herd (Mean ± SEM)^*^	14.2 ± 3.1^a^	26.9 ± 3.1^b^	32.9 ± 4.0^c^	12.2 ± 2.5^a^	21.5 ± 2.3

¶ Values in USD, average exchange rate during study = 3,620 UGX (2018–19). ^*^Total number of deaths from those under study, regardless of cause. SEM, standard error of mean. ^*^Different superscripts in rows shows mean values are statistically different (*p* < 0.05). Post-hoc analysis between groups was done using Tukey HSD (R package “multcomp”). ^a,b,c^Different superscripts within the same row implies mean values are statistically different.

In all, ninety-nine (99) out of 864 pigs raised from 94 farms (11.5%) were reported to have died during the study, whose perceived total value at farm-gate price before death was UGX 13,200,000 (USD 3,646.4). However, total mortalities reported were due to several causes, which were indistinguishable from each other.

### 3.1. Farm management standard scores (MSS)

At farm level, key factors known to influence occurrence of respiratory pathogens in herds include hygiene and biosecurity practices such as housing (influences hygiene, ventilation), floor type, isolation of sick pigs, access to extension services and routine use of dewormers and antibiotics (35, 36). Using above management variables, a score of farm management standard was derived, with a high score reflecting high management standards, while a lower score indicates low management standard. Herd size varied from 4.2 pigs for tethered pigs to 13.6 pigs per farm for housed pigs. About half of all sampled farms, 48.9% (*n* = 46) housed pigs, while the rest, 51.1% (*n* = 48) raised pigs by tethering near homesteads (Table 4).

**Table 4:** Summary of rearing method, herd size, and treatment costs per pig by management standard scores (MSS).

Variables	Management standard score (MSS)^*^		
	Poor (score 1)	Moderate (score 2)	High/better (score 3)
No. of farms which housed pigs, *n* (%)	0 (0.00%)	28 (29.80%)	18 (19.15%)
No. of farms which tethered pigs, *n* (%)	42 (44.68%)	6 (6.40%)	0 (0.00%)
Herd size (Mean ± SEM)^¶^	4.5 ± 0.23^a^	8.6 ± 0.70^b^	27.7 ± 2.85^c^
Treatment costs/pig (USD, Mean ± SEM)^¶^	1.13 ± 0.13^a^	0.92 ± 0.08^b^	0.95 ± 0.05^b^

^*^MSS was obtained as the sum of scores of house type, feed grade, isolation of sick pigs, access to extension services, routine use of drugs (antibiotics & dewormers), and pen hygiene. ^¶^Different superscripts in the last two rows (within row) show mean values are statistically different at *p* < 0.05. Post-hoc analysis between groups (farm scores) was done using Tukey HSD (R package “multcomp”).^a,b,c^Different superscripts within the same row implies mean values are statistically different.

The effect of management standard score on average treatment costs was demonstrated. Analysis of variance revealed significant differences in mean treatment costs per pig between MSS 3 and MSS 1 level farms (*F* value = 4.384, *p* = 0.012). High and moderate farm management standard farms showed significantly lower mean treatment costs compared to MSS 1 (poor) farms (Table 4). Table 5 shows a summary of live weights and proportions of pigs that tested positive in each visit.

**Table 5:** Summary statistics of live weights, PCV2, PRRSv, *M. hyo, App*, and helminths per visit.

Measure	Statistic	Visit number					
		1	2	3	4	5	
Live weight (kg)	Mean ± SD	25.6 ± 12.9	34.4 ± 14.3	44.0 ± 14.8	49.2 ± 17.7	54.0 ± 16.9	
ADG, gr/day	Mean ± SD	144.2 ± 80.2	154.98 ± 94.3	161.4 ± 93.4	168.1 ± 101.9	127.7 ± 58.8	
Proportion of pigs that tested positive to each pathogen per visit, *n* (%)							
Pathogen	Serostatus	Visit number					
		1	2	3	4	5	Totals
PCV2	Positive	15 (1.7)	26 (3.0)	24 (2.8)	20 (2.3)	3 (0.35)	88 (10.3)
	Negative	274 (32.0)	260 (30.4)	179 (20.9)	48 (5.6)	6 (0.7)	767 (89.7)
PRRSv	Positive	37 (4.3)	36 (4.2)	9 (1.0)	7 (0.8)	0 (0.0)	89 (10.4)
	Negative	252 (29.5)	250 (29.2)	195 (22.8)	61 (7.1)	8 (0.9)	766 (89.6)
*M. hyo*	Positive	16 (1.8)	17 (1.9)	13 (1.5)	5 (0.6)	0 (0.0)	51 (6.0)
	Negative	273 (31.9)	269 (31.5)	191 (22.3)	63 (7.3)	8 (0.9)	804 (94.0)
*App*	Positive	74 (8.6)	186 (21.7)	77 (9.0)	40 (4.6)	2 (0.2)	379 (44.3)
	Negative	215 (25.1)	100 (11.7)	127 (14.8)	28 (3.2)	6 (0.7)	476 (55.7)
*Ascaris spp*	Positive	26 (3.0)	14 (1.6)	13 (1.5)	6 (0.7)	0 (0.0)	59 (6.9)
	Negative	263 (30.7)	272 (31.8)	191 (22.3)	62 (7.2)	8 (0.9)	796 (93.1)
*Strongyles spp*	Positive	35 (4.0)	42 (4.9)	33 (3.8)	11 (1.3)	1 (0.1)	122 (14.3)
	Negative	254 (29.7)	244 (28.5)	171 (20.0)	57 (6.7)	7 (0.8)	733 (85.7)

### 3.2. Mixed effects model of ADG predictors with farm and pig as random effects

We fitted a linear mixed effects model to predict ADG with farm and pig as random effect terms (1 | Farm_ID) + (1 | Farm_ID: Pig_ID). A qqplot and a plot of residuals (distributed around mean zero) vs fitted values were used to verify the normality of data. Starting with a null model, predictors of ADG were added. Confounding was tested by inclusion and exclusion of variables and observing changes in model coefficients. Interactions between variables (e.g., age and sex) were tested but found non-significant and thus dropped from the final model. Based on AIC and BIC, the model (Table 6) provided the best fit to the data (*χ*^2^ = 17.20, *p* < 0.0001^***^). The mixed effects model showed infection with PPRSv and *Ascaris* spp. were marginally significant. Between-farm variance (Mean ± SD: 2636.2 ± 51.34) explained the greatest variation in ADGs (84.5%), followed by between-pig variance, 15.5% (Mean ± SD: 480.9 ± 21.93).

**Table 6:** Summary of a mixed effects model of predictors of ADG, with farm and pig as random effects.

Ind. variables	Estimate	95% Conf. Int	Std Err	*t* value	Pr(>|*t*|)
Intercept	119.50	89.36, 149.65	15.36	7.78	<0.001^**^
Age (months)	−6.58	−9.67, −3.49	1.57	−4.18	<0.001^**^
PRRSv infection	−17.10	−34.57, 0.38	8.90	−1.92	0.05.
Live weight (kg)	2.46	1.94, 2.99	0.27	9.18	<0.001^*^
*Ascaris spp.*	−16.80	−36.60, −3.06	10.10	−1.67	0.09
Feed grade	4.25	−6.16, 14.65	5.30	0.80	0.423
Mgt_level_2	−17.30	−38.81, 4.21	10.96	−1.58	0.115
Mgt_level_3	−2.85	−28.72, 23.02	13.1787	−0.22	0.829

Mgt_level: management standard score. 95% confidence intervals (CIs) and *p*-values were computed using a Wald *t*-distribution approximation. Reference values for age is 2 months, PRRSv is “0,” Live weight is 7.2 kg, Ascaris spp. is “0,” Feed grade is “1,” Management level is “1.” *p<0.05, **p<0.01.

Table 7 shows estimated monetary losses from ADG reductions associated with infections.

**Table 7:** Summary of estimated monetary losses from reductions in mean ADGs due to infections.

Pathogen/management level	Mean ADG reduction (gr/pig/day)	Weight loss (gr) during fattening (200 days)	Est. carcass dressing %^§^	Mean weight loss (kg)	Total monetary losses per pig (UGX)	Monetary losses/pig (Mean ± SEM)^*^
PRRSv	17.10	3,420	70	2.40	24,000	6.6 ± 2.7
*Ascaris spp.*	16.80	3,360	70	2.35	23,520	6.5 ± 3.2
*Mgt_level_2*	17.30	3,460	70	2.42	24,200	6.7 ± 3.6

^*^Estimated monetary losses are presented in USD. Average exchange rate during the study, ^*^USD = 3,620 UGX (2018–2019); average days of fattening in Uganda (200 days, from 2–8.5 months); ^§^Carcass dressing percentage was obtained from Kugonza et al. (37) for pigs on maize bran-based diet. Average price/kg of pork during study (UGX 10,000, equivalent to USD 2.76), SEM is standard error of mean.

## 4. Discussion

This study highlights the role of farm management and the impacts of respiratory infections on pig daily weight gains in Uganda. To support farm decision making, a clear understanding of farm management practices and their relationship with weight gains and production costs is required. Ultimately, the goal of any producer is to minimize production costs, reduce or eliminate economic losses, which translate into better profit margins. These findings compare favorably with other studies elsewhere, which reveal negative effects of respiratory diseases on daily weight gains (38, 39), increased financial expenditures (1, 6), and reduced profit margins (40). A recent study which reported high prevalence of pneumonia lesions in slaughtered pigs highlights the likely contribution of respiratory infections to lung pathology (41).

Our findings align with other studies elsewhere, which reported a drop in mean ADGs with increase in respiratory disease prevalence (39). In this study, pigs within the same farm and observations within pig differed significantly with pathogen exposure status. While pigs exposed to PRRSv and *Ascaris* spp. gained less (marginally significant) ADGs compared to those unexposed (Table 6), it suggests adverse effects of pathogens on weight gains. As expected, age was a significant predictor of ADGs, but showed a negative association with ADGs. This is perhaps due the observation that pigs tended to gain less weight as they aged, as shown by wide variations in live weights relative to age.

These findings are consistent with other studies which reported a reduction in ADGs by between 8 and 14%, and increased mortality of 19.9% in farms with a high disease challenge (5). A study reported a reduction in ADGs of between 16 and 29% for respiratory and between 8.4 and 19.4% for parasitic infections (42). The mean ADG of pigs reported in this study compares to that in other studies in East Africa in similar settings. In Uganda, a study reported that the ADG of nursery pigs fed on forage-based diet was 160 gr/day (43), while another recent study in Lira district reported 101 gr/day (14). In Western Kenya, (25) reported ADG of 130 gr/pig/day, while in Tanzania, Lipendele and colleagues reported ADG of 136 gr/pig/day (44). However, these ADG values are generally much lower compared to those in other countries, which attain 600 gr/day or higher (5, 39).

The low ADGs of pigs in this study (compared to that in developed economies) could be explained by endemic infections, underfeeding and inferior genetics as reported in recent studies in Uganda (14, 43, 45). The adverse effects of mixed infections on ADGs confirm findings from previous studies which showed mixed infections reduced ADGs, led to more severe and prolonged duration of respiratory disease (10, 46). The effect of infective dose, pathogen type and strains involved, their interactions with environmental stressors and the subsequent response of the pig’s immune system play a significant role in the induction of clinical disease. These interactions lead to subclinical, mild, or severe disease outcome, producing varying effects on weight gains and other productive indices as previously reported (47, 48).

This study revealed that mean treatment costs declined with improvement in management standard score. Farms with a high level of management (*MSS* 3) spent significantly lower mean treatment costs compared to those with poor (*MSS* 1) management level (Table 4). This is unsurprising, since farms with poor management were reported to have higher disease incidence in previous studies (39, 49). These findings suggest farmers could make substantial monetary savings from adverse disease impacts if they adopted better management practices, i.e., move from level two to level three MSS. These include improved housing, hygiene and biosecurity measures, better nutrition, and regular deworming of pigs.

In this study, we show that monetary losses per pig associated with PRRSv and *Ascaris* spp. infections were substantial for smallholder farmers. These estimates are quite conservative and likely represent a fraction of potential total losses encountered as other productivity indices (e.g., abortions, mortalities) were not captured. In this study, partly due to underfeeding, farmers often kept pigs for longer than 200 days (6.5 months), which adds to possible losses from extra feeds needed to raise pigs to market weight. In the US, (5) reported that financial costs under commercial conditions in high disease challenge farms varied between USD 8.5 and 29.8 per marketed pig, while Dee and Joo (50) reported costs due to PRRSv infection between 2005 and 2010 ranged from 10.5 to 12.5 USD per marketed pig. However, these studies were done in intensive, high-efficiency settings, in contrast to smallholder production systems in our study.

Housing type and quality have a direct and indirect effect on pathogen transmission between pigs. Floor types (deep litter, elevated timber platform, cement, and rammed soil/murram) influences pig welfare and hygiene. Farmer attitude and behaviour determines the frequency with which wastes are removed from pens. These management factors directly influence the pathogen load that may accumulate and multiply in pens, particularly if the floor was poorly designed. Accumulation of pathogens in pens due to lack of cleaning may facilitate transfer of infection(s) between pigs. Cargill (35) reported that pigs reared in an all-in-all-out (AIAO) system with cleaning grew by 15% faster than pigs reared with no cleaning. The same study showed that pigs on dry floors gained higher ADGs (5% higher) than pigs on wet floors. A similar finding was reported in a study by Pastorelli et al., which found that pigs raised in poor sanitary conditions gained 11% significantly lower ADG compared to those under good sanitary conditions (42). Evidence of the role of good hygiene in pig health, overall welfare, and efficiency of the value chain, generating better financial returns to the producer is documented (1). Our study provides a framework to measure the quality of pig housing and management level in smallholder settings, both of which influence health, welfare and productivity. It can be adapted to a given context to include other management practices (e.g., beddings), which most farmers did not provide.

The fact that only a third of farmers in this study had access to extension services justifies a necessity to strengthen these services to provide technical advice on herd health and biosecurity. While prophylactic use of antibiotics against bacterial pathogens is known to reduce the burden of opportunistic infections (51, 52), their judicious use should be promoted to guard against possible misuse, which could escalate the problem of antimicrobial resistance (AMR). The practice of isolating sick pigs helps minimize the risk of further pathogen spread between pigs. These herd preventive practices are critical as they influence the level of contamination, risk and extent of pathogen spread between pigs. A study showed routine management factors (e.g., routine removal of manure) had a greater impact on *Ascaris suum* infection than regular deworming (53). This underscores the importance of “*good*” management in reducing adverse effects of disease. Management is a combination of a farmer’s socio-economic attitude, behaviour and practices, which reflects their skills and knowledge of possible disease impacts on herd performance. However, farmers’ adoption of biosecurity measures for disease control should be supported by incentives that increase their financial returns, as previously highlighted (54). The observation that farmers’ education level directly correlated with management level underscores the importance of education in reducing adverse disease impacts. It is therefore important to consider the social context when designing health management interventions.

This study was limited to estimation of direct costs due to respiratory and worm infections. However, it was impossible to estimate other indirect economic costs (specific to respiratory diseases) attributable to deaths and salvage prices of sick pigs due to lack of reliable farm data. That these indirect costs were not captured suggests economic losses encountered by farmers in this study may have been underestimated. Besides, because not all farmers treated their pigs despite showing clinical disease due to lack of cash or no access to extension worker, it’s probable that errors in estimates of treatment costs may have been introduced. Because ELISA tests used reflect prior exposure of pigs to respiratory pathogens, knowledge gaps remain on the duration of infection(s), and the time lapse to induce clinical disease during individual or mixed infections, both of which ultimately influence growth rates.

## 5. Conclusions and recommendations

To the best of our knowledge, this is the first study in Uganda to document evidence of adverse effects of respiratory and helminth infections on weight gains in pigs. We showed that a grower pig in a given farm exposed to PRRSv and *Ascaris* spp. infection had significantly lower daily weight gains (ADG) by 17.10 gr/day and 16.80 grams/day respectively, compared to a similar unexposed pig of the same age. Mean treatment costs per pig declined with improvement in management level scores (MLS), from USD 1.13 in MLS 1 (poor management) farms to USD 0.95 per pig for MLS 3 (better management) farms. We show that monetary losses encountered by farmers due to PRRSv and *Ascaris spp.* infection amounted to USD 6.60 ± 2.7 and 6.50 ± 3.2 (Mean ± SEM) per pig, respectively during 200 days of fattening.

This study highlights the role good management plays in mitigating against adverse effects of respiratory infections in pigs. To reduce possible economic losses from disease, it is important for farmers to adopt good herd management practices using welfare concept, which include proper housing, nutrition, and biosecurity. These prerequisites are necessary for optimal growth and health of pigs, which enhances farm profitability. Further studies are required to establish the full extent of other possible indirect losses (e.g., reproductive disorders) considering pathogen interactions and variations in disease severity.

## Data availability statement

The original contributions presented in the study are included in the article/supplementary material, further inquiries can be directed to the corresponding author.

## Ethics statement

The animal study was reviewed and approved by ILRI’s Institutional Research Ethics Committee (IREC no. IREC2018-23) ILRI’s Institutional Animal Care and Use Committee (IACUC2018-22) Institutional Review Board (IRB no. SBLS/REC/18/008), College of Veterinary Medicine, Animal Resources and Biosecurity (CoVAB), Makerere University. Written informed consent was obtained from the owners for the participation of their animals in this study.

## Author contributions

MD, PO, JE, FM, BW, and EO designed the study. PO performed data collection. PO, MD, and EO analyzed data. PO and EO prepared draft manuscript. All authors contributed to the article and approved the submitted version.

## Funding

We acknowledge funding support from the CGIAR Research Programme on Livestock and the OneCGIAR initiative “*Sustainable Animal Productivity for Livelihoods, Nutrition and Gender (SAPLING) inclusion*” (https://www.cgiar.org/initiative/17-sustainable-animal-productivity-for-livelihoods-nutrition-and-gender-inclusion-sapling/) and all donors and organizations which globally support its work through their contributions to the CGIAR Trust Fund https://www.cgiar.org/funders/trust-fund/. The support received from the German Academic Exchange (DAAD) Service (Personal ref. no 91672538) for Peter Oba’s PhD programme at Makerere University is acknowledged.

## Conflict of interest

The authors declare that the research was conducted in the absence of any commercial or financial relationships that could be construed as a potential conflict of interest.

## Publisher’s note

All claims expressed in this article are solely those of the authors and do not necessarily represent those of their affiliated organizations, or those of the publisher, the editors and the reviewers. Any product that may be evaluated in this article, or claim that may be made by its manufacturer, is not guaranteed or endorsed by the publisher.

## References

[1] Calderón Díaz JA Fitzgerald RM Shalloo L Rodrigues da Costa M Niemi J Leonard FC (2020). Financial analysis of herd status and vaccination practices for porcine reproductive and respiratory syndrome virus, swine influenza virus, and *Mycoplasma hyopneumoniae* in farrow-to-finish pig farms using a bio-economic simulation model. Front Vet Sci 7:1–14. doi: 10.3389/fvets.2020.556674, PMID: 33240946 PMC7680737

[2] Ferraz M Almeida H Storino G Sonálio K Souza M Moura C (2020). Lung consolidation caused by *Mycoplasma hyopneumoniae* has a negative effect on productive performance and economic revenue in finishing pigs. Prev Vet Med 182:105091. doi: 10.1016/j.prevetmed.2020.105091, PMID: 32683190

[3] Nieuwenhuis N Duinhof T van Nes A (2012). Economic analysis of outbreaks of porcine reproductive and respiratory syndrome virus in nine sow herds. Vet Rec 170:225. doi: 10.1136/vr.100101, PMID: 22238201

[4] Alarcon P Rushton J Nathues H Wieland B (2013). Economic efficiency analysis of different strategies to control post-weaning multi-systemic wasting syndrome and porcine circovirus type 2 subclinical infection in 3-weekly batch system farms. Prev Vet Med 110:103–18. doi: 10.1016/j.prevetmed.2012.12.006, PMID: 23375866 PMC3652493

[5] Cornelison AS Karriker LA Williams NH Haberl BJ Stalder KJ Schulz LL (2018). Impact of health challenges on pig growth performance, carcass characteristics, and net returns under commercial conditions. Transl Anim Sci 2:50–61. doi: 10.1093/tas/txx005, PMID: 32289106 PMC7107292

[6] Nathues H Alarcon P Rushton J Jolie R Fiebig K Jimenez M (2017). Cost of porcine reproductive and respiratory syndrome virus at individual farm level – an economic disease model. Prev Vet Med 142:16–29. doi: 10.1016/j.prevetmed.2017.04.006, PMID: 28606362

[7] Opriessnig T Thacker E Yu S Fenaux M Meng X Halbur G (2004). Experimental reproduction of postweaning multisystemic wasting syndrome in pigs by dual infection with mycoplasma hyopneumoniae and porcine circovirus type 2. Vet Pathol 41:624–40. doi: 10.1354/vp.41-6-624, PMID: 15557072

[8] Brombilla T. Ogata R. A. Nassar A. F. De C. Cardoso M. V. Ruiz V. L. De A., and Fava C. Del (2019). Effect of bacterial agents of porcine respiratory disease complex on productive indices and slaughter weight. Ciência Anim Bras 20, 1–12. doi: 10.1590/1809-6891v20e-51615

[9] Scollo A Gottardo F Contiero B Mazzoni C Leneveu P Edwards SA (2017). Benchmarking of pluck lesions at slaughter as a health monitoring tool for pigs slaughtered at 170 kg (heavy pigs). Prev Vet Med 144:20–8. doi: 10.1016/j.prevetmed.2017.05.007, PMID: 28716200

[10] Opriessnig T Halbur PG (2012). Concurrent infections are important for expression of porcine circovirus associated disease. Virus Res 164:20–32. doi: 10.1016/j.virusres.2011.09.014, PMID: 21959087 PMC7114432

[11] Thacker E Halbur P Ross R Thanawongnuwech R Thacker B (1999). *Mycoplasma hyopneumoniae* potentiation of porcine reproductive and respiratory syndrome virus-induced pneumonia. J Clin Microbiol 37:620–7. doi: 10.1128/JCM.37.3.620-627.1999, PMID: 9986823 PMC84495

[12] Chenais E Sternberg-Lewerin S Boqvist S Liu L LeBlanc N Aliro T (2017). African swine fever outbreak on a medium-sized farm in Uganda: biosecurity breaches and within-farm virus contamination. Trop Anim Health Prod 49:337–46. doi: 10.1007/s11250-016-1197-0 27966070 PMC5253150

[13] Dione M Masembe C Akol J Kungu J Amia W Wieland B (2016). Occurrence of selected bacterial and viral pathogens in smallholder pig production systems in Uganda 3:2016. 10.1016/j.actatropica.2018.06.025 29949731

[14] Gertzell E Magnusson U Ikwap K Dione M Lindström L Eliasson-Selling L (2021). Animal health beyond the single disease approach – a role for veterinary herd health management in low-income countries? Res Vet Sci 136:453–63. doi: 10.1016/j.rvsc.2021.03.021, PMID: 33812288

[15] Ouma E Ochieng J Dione M Pezo D (2017). Governance structures in smallholder pig value chains in Uganda: constraints and opportunities for upgrading. Int Food Agribus Manag Rev 20:307–19. doi: 10.22434/IFAMR2014.0176

[16] UBOS (2014). Uganda bureau of statistics statistical abstract. 1–305. Available at: http://www.ubos.org/onlinefiles/uploads/ubos/statistical_abstracts/StatisticalAbstract 2014.pdf

[17] Atuhaire D Ochwo S Afayoa M Norbert Mwiine F Kokas I Arinaitwe E (2013). Epidemiological overview of African swine fever in Uganda (2001–2012). J Vet Med 2013:1–9. doi: 10.1155/2013/949638, PMID: 26464916 PMC4590872

[18] Muhangi D Masembe C Berg MKS Ocaido M (2014). Practices in the pig value chain in Uganda; implications to African swine fever transmission. Livest Res Rural Dev 26:18. Available at: http://www.lrrd.org/lrrd26/5/muha26094.htm

[19] Dione M Masembe C Akol J Amia W Kungu J Lee HS (2018). The importance of on-farm biosecurity: Sero-prevalence and risk factors of bacterial and viral pathogens in smallholder pig systems in Uganda. Acta Trop 187:214–21. doi: 10.1016/j.actatropica.2018.06.025, PMID: 29949731

[20] Eneku W Mutebi F Mwiine F Okwee-Acai J Ojok L (2018). Porcine circovirus type 2 – systemic disease on pig farms and associated knowledge of key players in the pig industry in Central Uganda. Int J Vet Sci Med 6:178–85. doi: 10.1016/j.ijvsm.2018.08.004, PMID: 30564593 PMC6286401

[21] Jonsson L. (2013). Emerging Infectious diseases: Using PCV2 as a model of disease transmission dynamics at the livestock-wildlife interface in Uganda, Swedish University of Agricultural Sciences, Faculty of Veterinary Medicine and Animal Science.

[22] Ouma E. Overview of ILRI’s smallholder pig value chain efforts in Lira district. Uganda: Hoima (2017).

[23] Ikwap K Jacobson M Lundeheim N Owiny D Nasinyama GW Fellstrom C (2014). Characterization of pig production in Gulu and Soroti districts in northern and eastern Uganda. Livest Res Rural Dev 26, 1–9. Available at: https://lrrd.cipav.org.co/lrrd26/4/ikwa26074.htm

[24] Kungu JM Masembe C Apamaku M Akol J Amia WC Dione M (2019). Pig farming systems and cysticercosis in northern Uganda. Rev d’élevage médecine vétérinaire des pays Trop 72:115–21. doi: 10.19182/remvt.31254

[25] Carter N Dewey C Mutua F de Lange C Grace D (2013). Average daily gain of local pigs on rural and Peri-urban smallholder farms in two districts of Western Kenya. Trop Anim Health Prod 45:1533–8. doi: 10.1007/s11250-013-0395-2, PMID: 23504593

[26] Dohoo I Martin W Stryhn H. Veterinary epidemiologic research. 2nd ed. Charlottetown, Prince Edward Island, Canada: VER, Incorporated (2003).

[27] Carter N Dewey CE Lukuyu B Grace D de Lange C (2015). Nutritional value and seasonal availability of feed ingredients for pigs in Uganda. Agric Trop Subtrop 48:91–104. doi: 10.1515/ats-2015-0013

[28] Jayasinghe P Ramilan T Donaghy DJ Pembleton KG Barber DG (2022). Comparison of nutritive values of tropical pasture species grown in different environments, and implications for livestock methane production: a meta-analysis. Animals 12:1806. doi: 10.3390/ani12141806, PMID: 35883354 PMC9311783

[29] Oba P Dione M Erume J Wieland B Mutisya C Ochieng L (2022). Molecular characterization of porcine reproductive and respiratory syndrome virus (PRRSv) identified from slaughtered pigs in northern Uganda. BMC Vet Res 18:176–8. doi: 10.1186/s12917-022-03272-x, PMID: 35562693 PMC9102683

[30] MAFF (1986). Manual of veterinary parasitological laboratory techniques. 3rd. London: H.M.S.O. Print. Reference book: Great Britain. Ministry of Agriculture, fisheries and food.

[31] R Core Team. R: A language and environment for statistical computing. Vienna, Austria: R Foundation for Statistical Computing (2019) Available at: https://www.r-project.org/.

[32] Mutua FK Dewey CE Arimi SM Schelling E Ogara WO (2011). Prediction of live body weight using length and girth measurements for pigs in rural Western Kenya. J Swine Heal Prod 19:26–33. Available at: http://www.aasv.org/shap/issues/v19n1/v19n1p26.html

[33] Bates D Mächler M Bolker B Walker S (2015). Fitting linear mixed-effects models using lme4. J Stat Softw 67:1–48. doi: 10.18637/jss.v067.i01

[34] Njuki J. Poole J. Johnson N. Baltenweck I. Pali P. Mburu S. (2011). Gender, Livestock and Livelihood Indicators. Nairobi, Kenya: International Livestock Research Institute (ILRI).

[35] Cargill C. The impact of environment on production and health. In regional symposium on research into smallholder pig production, health, and pork safety. Hanoi, Vietnam: ILRI (2019).

[36] Stärk K. (1998). Systems for the prevention and control of INFECTIOUS diseases in pigs. Palmerston North, New Zealand: Massey University.

[37] Kugonza D. R. Lubandi C. Kirembe G. Taabu H. L. Mugala L. K. Lusembo P. (2017). Enhancing pig productivity in the Lake Victoria crescent zone through genotype and Postweaning diet interventions, Kampala, Uganda: Book of NARO/Mak Conference Abstracts. 277–287.

[38] Agostini P Fahey A Manzanilla E O’Doherty J de Blas C Gasa J (2014). Management factors affecting mortality, feed intake and feed conversion ratio of grow-finishing pigs. Animal 8:1312–8. doi: 10.1017/S1751731113001912 24229728

[39] Gray H Friel M Goold C Smith RP Williamson SM Collins LM (2021). Modelling the links between farm characteristics, respiratory health and pig production traits. Sci Rep 11:13789–13. doi: 10.1038/s41598-021-93027-9, PMID: 34215759 PMC8253804

[40] Renken C Nathues C Swam H Fiebig K Weiss C Eddicks M (2021). Application of an economic calculator to determine the cost of porcine reproductive and respiratory syndrome at farm-level in 21 pig herds in Germany. Porc Heal Manag 7:3–12. doi: 10.1186/s40813-020-00183-x, PMID: 33397503 PMC7784293

[41] Oba P Dione M Wieland B Mwiine F Erume J (2021). Correlations between lung pneumonic lesions and serologic status for key respiratory pathogens in slaughtered pigs in northern Uganda. Porc Heal Manag 7:53–10. doi: 10.1186/s40813-021-00233-y, PMID: 34607613 PMC8489042

[42] Pastorelli H Van Milgen J Lovatto P Montagne L (2012). Meta-analysis of feed intake and growth responses of growing pigs after a sanitary challenge. Animal 6:952–61. doi: 10.1017/S175173111100228X, PMID: 22558966

[43] Carter N Dewey C Grace D Lukuyu B Smith E de Lange C (2016). Average daily gain and the impact of starting body weight of individual nursery and finisher Ugandan pigs fed a commercial diet, a forage-based diet, or a silage-based diet. J Swine Heal Prod 25:121–8. Available at: https://www.aasv.org/shap/issues/v25n3/v25n3p121.html

[44] Lipendele CP Lekule FP Mushi DE Ngowi H Kimbi EC Mejer H (2015). Productivity and parasitic infections of pigs kept under different management systems by smallholder farmers in Mbeya and Mbozi districts. Tanzania Trop Anim Heal Prod 47:1121–30. doi: 10.1007/s11250-015-0836-1, PMID: 25934145

[45] Lukuyu B. Lule P. Kawuma B. Ouma E. (2017). Feeds and forage interventions in the smallholder pig value chain of Uganda. ILRI Research Brief 78. 5.

[46] Niederwerder M Jaing C Thissen J Cino-Ozuna A McLoughlin K Rowland R (2016). Microbiome associations in pigs with the best and worst clinical outcomes following co-infection with porcine reproductive and respiratory syndrome virus (PRRSV) and porcine circovirus type 2 (PCV2). Vet Microbiol 188:1–11. doi: 10.1016/j.vetmic.2016.03.008, PMID: 27139023

[47] Alarcon P Velasova M Mastin A Nevel A Stärk K Wieland B (2011). Farm level risk factors associated with severity of post-weaning multi-systemic wasting syndrome. Prev Vet Med 101:182–91. doi: 10.1016/j.prevetmed.2011.06.001, PMID: 21741715

[48] Brockmeier SL Palmer MV Bolin SR Rimler RB (2001). Effects of intranasal inoculation with *Bordetella bronchiseptica*, porcine reproductive and respiratory syndrome virus, or a combination of both organisms on subsequent infection with *Pasteurella multocida* in pigs. Am J Vet Res 62:521–5. doi: 10.2460/ajvr.2001.62.521, PMID: 11327458

[49] Merialdi G Dottori M Bonilauri P Luppi A Gozio S Pozzi P (2012). Survey of pleuritis and pulmonary lesions in pigs at abattoir with a focus on the extent of the condition and herd risk factors. Vet J 193:234–9. doi: 10.1016/j.tvjl.2011.11.009 22182431

[50] Dee S. Joo H. (1994). Factors involved in successful eradication of PRRS virus using nursery depopulation. AASV Annual Meeting, 239–243.

[51] Adedeji SO Ogunba EO Dipeolu OO (1989). Synergistic effect of migrating Ascaris larvae and *Escherichia coli* in piglets. J Helminthol 63:19–24. doi: 10.1017/S0022149X00008671, PMID: 2656846

[52] Steenhard N Jungersen G Kokotovic B Beshah E Dawson H Urban J (2009). Ascaris suum infection negatively affects the response to a *Mycoplasma hyopneumoniae* vaccination and subsequent challenge infection in pigs. Vaccine 27:5161–9. doi: 10.1016/j.vaccine.2009.05.075, PMID: 19524617

[53] Roesel K Dohoo I Baumann M Dione M Grace D Clausen PH (2017). Prevalence and risk factors for gastrointestinal parasites in small-scale pig enterprises in central and eastern Uganda. Paras Res 116:335–45. doi: 10.1007/s00436-016-5296-7 PMC5167772 27785599

[54] Ouma E Dione M Birungi R Lule P Mayega L Dizyee K (2018). African swine fever control and market integration in Ugandan Peri-urban smallholder pig value chains: an ex-ante impact assessment of interventions and their interaction. Prev Vet Med 151:29–39. doi: 10.1016/j.prevetmed.2017.12.010, PMID: 29496103

